# Stress Analysis of Endodontically Treated Tooth–Implant Different Connectors Designs in Maxillary Posterior Region: A Finite Element Analysis

**DOI:** 10.1055/s-0043-1772780

**Published:** 2023-10-17

**Authors:** Sara Hashemi, Kimia Baghaei, Amirhossein Fathi, Navid Aghadavoudi, Seyed Saman Hashemi, Ramin Atash, Sayed Sobhan Khademi

**Affiliations:** 1 Dental Students Research Committee, School of Dentistry, Isfahan University of Medical Sciences, Isfahan, Iran; 2 Department of Dental Prosthodontics, Dental Materials Research Center, School of Dentistry, Isfahan University of Medical Sciences, Isfahan, Iran; 3 Private Practice in Isfahan, Iran; 4 Department of Prosthodontics, School of Dentistry, Faculty of Medicine, Université Libre de Bruxelles, Brussels, Belgium; 5 Department of Prosthodontics, School of Dentistry, Islamic Azad University (Khorasgan Branch), Isfahan, Iran

**Keywords:** finite element, fixed prostheses, implant, maxillary, postcrown, tooth

## Abstract

**Objectives** Using finite element analysis (FEA), this study aimed to determine the effect of nonrigid connectors (NRCs) and their position on the success of tooth and implant-supported fixed prostheses in the maxillary posterior region.

**Materials and Methods** Three three-dimensional FEA models were designed, presuming maxillary second premolar and first molar to be extracted. Implant (replacing first molar), abutment, bone (spongious and cortical), first premolar (containing dentin, root cement, gutta-percha, and casting post and core), periodontal ligament, and three three-unit cemented porcelain-fused-to-metal prostheses (a rigid one and two nonrigid) were modeled. The NRC was once on the tooth side and once on the implant side. The prostheses were loaded twice. The first molar (180 N) and premolars (120 N) teeth were subjected to progressive vertical and oblique (12-degree) loads, and maximum von Mises stress and strain in teeth and connectors were calculated for each model.

**Results** The findings of the current study showed evidence that tooth-implant design with an NRC has significantly increased the average stress in the tooth. The average stress in dentin was 769.02 for the mesial connector and 766.95 for the distal connector, and this was only 731.59 for rigid connector. Furthermore, it was observed that rigid connector has considerably minimized the stress within the tooth–implant-supported fixed partial denture. The average stress for the crown and metal frame is 346.22 and 526.41 in rigid connector, while it is 1,172.9 and 2,050.9 for the nonrigid mesial connector.

**Conclusion** Although distal NRC was more efficient than mesial NRC, using NRC will only reduce the stress applied to cortical bone and is not recommended in the posterior region of the maxilla.

## Introduction

Dental implants have been accepted by clinicians because of the long-term survival rate for oral rehabilitation in partially or complete edentulous patient. 1 Additionally, patients prefer fixed prostheses in comparison to removable dentures because they provide higher quality of life. 2 However, having an implant-supported fixed prosthesis is not always available due to anatomic (e.g., insufficient available bone) or economic issues or the loss of osseointegration. 3 4 Considering the increase of remaining teeth in elderly and fixed prosthesis benefits, a tooth and implant-supported fixed prosthesis (TIFP) may be considered as a treatment plan option. 5 6

Since the early 1980s, the teeth were combined with implants to support fixed dental prostheses. 7 But there was always a controversy among clinicians about connecting an implant to natural tooth. 8 According to Branemark protocol, TIFPs should be avoided. 9 Biomechanical challenges are the main reasons for this debate. Since natural teeth are surrounded by periodontal ligament, they have a mobility of 50 to 200 μm but a healthy bone structure only allows 10 μm movement for implants. 10 Additionally, natural teeth have a rapid movement and a linear one, whereas implants only have the linear movement. 11 The difference between teeth and implant movement leads to different patterns of stress and strain in the tooth, implant, and the surrounding structure, When an occlusal force is applied to a joined natural tooth–implant, the stress does not evenly distribute in the prosthesis resulting in a destructive load on the implant, abutments, and the entire prosthesis. 12 Additionally, complications such as an increased incidence of caries at the crown margin, tooth intrusion, and mechanical part fracture have been described. To minimize these effects, different designs of rigid connectors, nonrigid connectors (NRCs), and implant-absorbing elements have been suggested to compensate for the difference in the amount of settlement between natural teeth and implants. 13 14

Several studies raised the concern of tooth intrusion due to using a NRC and suggested a rigid connector. 15 16 However, others suggested that connector type has no significant effect on tooth intrusion. 17 18 While several finite element analysis (FEA) studies have shown that using a flexible joint system, the NRC, can modify the stress distribution among the tooth and the implant under axial force, preventing undue load on the implant-supporting bone, 19 20 21 22 some of the findings about different connection designs of TIFPs in distal extension situations using FEA reported that using a rigid connector or NRC in the natural tooth or implant side of the pontic has no significant effect. 23 There are controversial results in clinical surveys regarding the advantages of NRC versus rigid connectors. 24

As far as the authors are concerned, all the available data on this topic analyzed forces applied to mandibular teeth, while occlusal forces on the working sides are applied in different directions in maxillary and mandibular regions. 25 Furthermore, the vital teeth restored with crowns as part of a fixed partial denture have a high risk of pulpal necrosis, it is more accurate to simulate the tooth, which plays a role as a pier abutment, in an endodontically treated situation rather than a vital tooth due to their different characteristics. Surprisingly, most of the previous FEA studies used natural teeth. Natural tooth, gutta-percha, and post materials show different physical characteristics and behaviors under occlusal forces. 26 This study aims to clarify the effect of the presence and location of NRCs on the stress distribution on the TIFPs in the maxillary posterior region in two different occlusal schemes by means of FEA.

## Materials and Methods

### Modeling in Mimix and 3-matic Software and Format Conversion

Converge analysis check was performed to choose the optimal size and number of elements ( Appendix A ). To model a partially edentulous maxilla with the second premolar and first molar have been extracted and the existing first premolar tooth with two separate roots and canals, computed tomography scan photos with a distance of 1 mm between the slices ( Appendix B ) were imported into three-dimensional (3D) image processing software (Mimics and 3-matic software, Mimics Research 21; Materialise NV; Brussel, Belgium) and models of bones (cortical and spongy), teeth, periodontal ligament, and root cement were modeled and then prepared in 3D form. Posterior maxillary ridge height was assumed to be 13 to 16 mm, the cortical bone thickness was 1.5 mm, and the periodontal membrane was considered to be 0.3 mm thick ( Appendix C ). Later, abutment and implant models were made in SolidWorks 2020 software using Helix commands and Surface creation ( Appendix D ). As a result, a Straumann bone level tapered implant with a diameter of 4.1 mm and a height of 10 mm and a cement-retained Straumann CARES titanium abutment with a gingival height of 1.5 mm were designed and placed in the maxillary first molar location ( Appendix D ). The implant was assumed to be 100% osseointegrated. The maxillary first premolar was considered to be endodontically treated, 4 mm of the apical part of both of the canals was filled with gutta-percha, and a casting post and core of nickel–chromium (Ni-Cr) alloy were designed for the tooth with 2 mm of the ferrule, which was cemented by zinc phosphate cement. The tooth was conventionally prepared for a porcelain-fused-to-metal (PFM) restoration. The preparation of natural teeth and the creation of metal–ceramic restorations were done as per prosthetic guidelines. 9 The 0.5 mm thickness Ni–Cr alloy core was veneered with 1.5-mm thickness porcelain. Finally, porcelain was cemented to the tooth and implant by zinc phosphate cement and modeled as three three-unit PFM restorations with three different connector sets. The design of the first implant restoration and premolar is shown in Appendix E . Then, the different NRCs were designed. In the first condition, both connectors are rigid (original design); in the second, the connector on the mesial side is nonrigid and the distal one is rigid; and in the third, the connector connected to the implant is nonrigid and the other is rigid. The NRC was placed with deep preparation. Slide-type attachment (T-123, Metalor, Neuchatel, Switzerland) was the NRC ( Appendix F ). The NRC was 2 mm in length in the vertical plane for all the models. Afterward, all the parts were exported as .stl format from this software and converted to .stp format using Geomagic software (Geomagix Design X; Geomagic Inc., Rock Hill, South Carolina, United States). Contact conditions for every contact body were considered bonded or tied. The design of the first implant restoration and premolar is shown in Appendix G .

**Appendix A:**
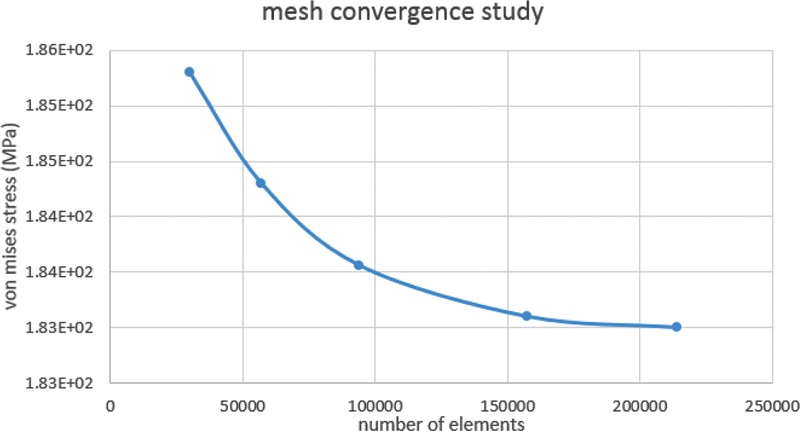
Converge analysis check.

**Appendix B:**
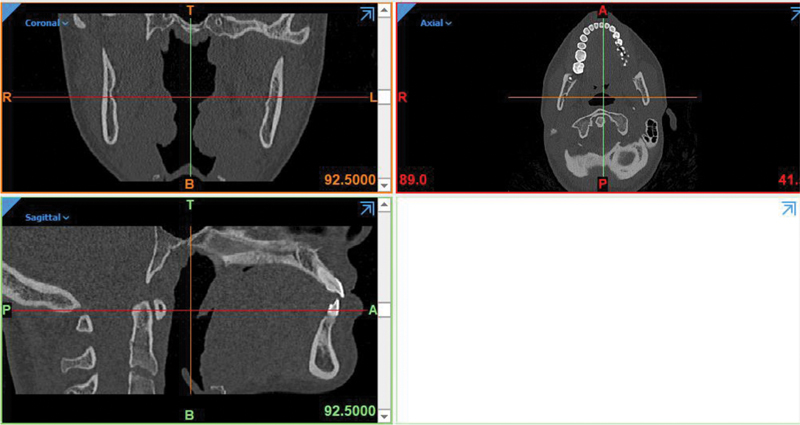
(1) Modeling in Mimic and 3-matic software. The models of bones, teeth, periodontal ligament, and cement were modeled in Mimics and Trimetic software.

**Appendix C:**
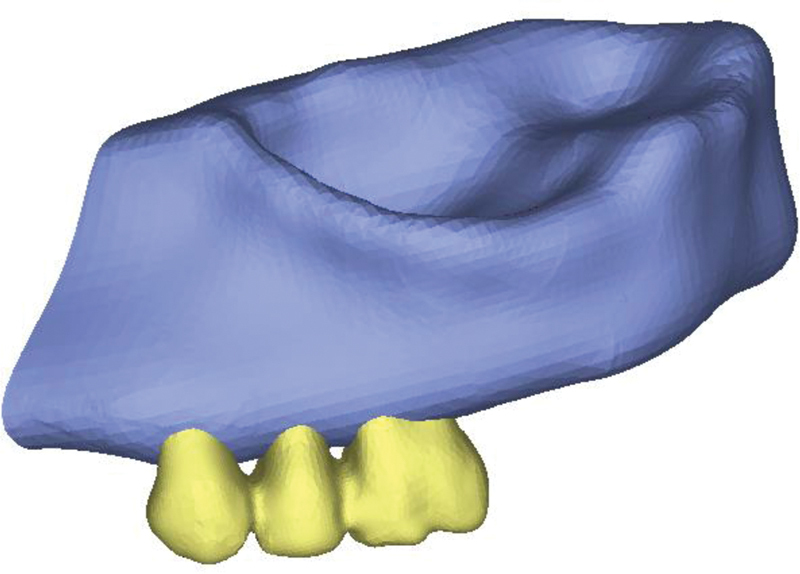
3D models of component.

**Appendix D:**
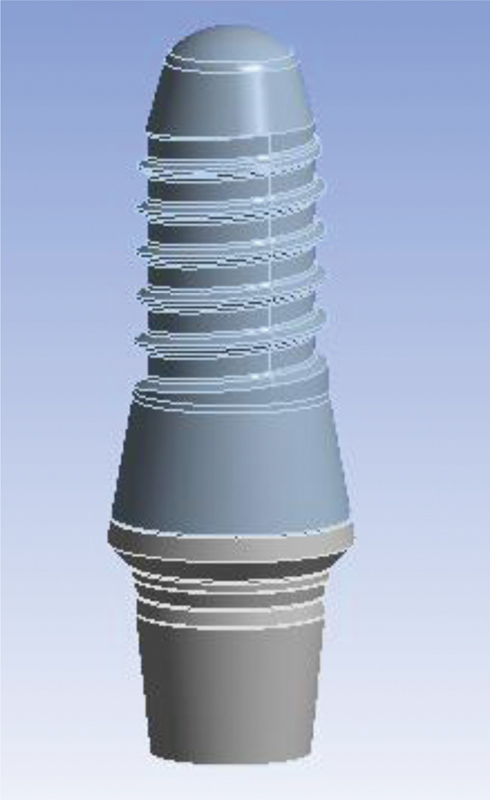
Implant modeling in stl.

**Appendix E:**
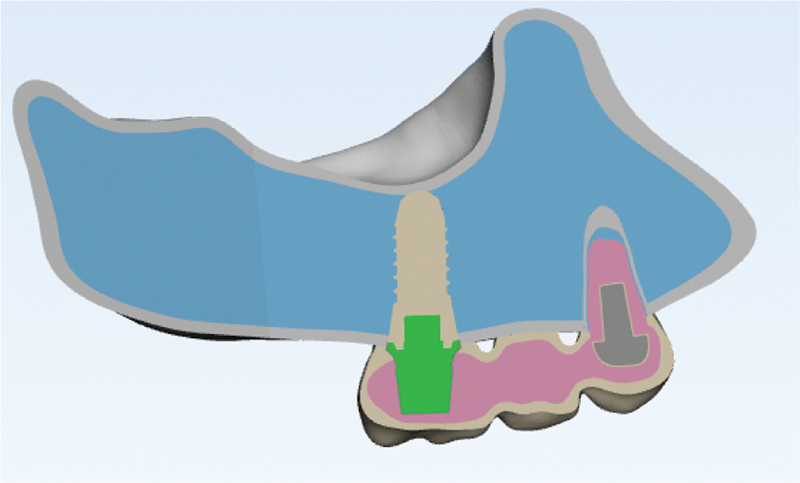
Design of first implant restoration and premolar.

**Appendix F:**
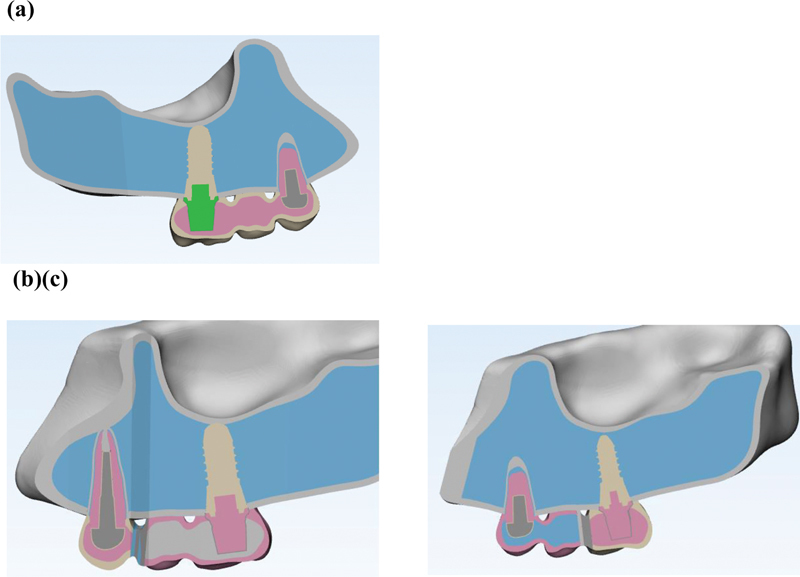
Connectors design (a) rigid connector, (b) mesial nonrigid connector, (c) distal nonrigid connector.

**Appendix G:**
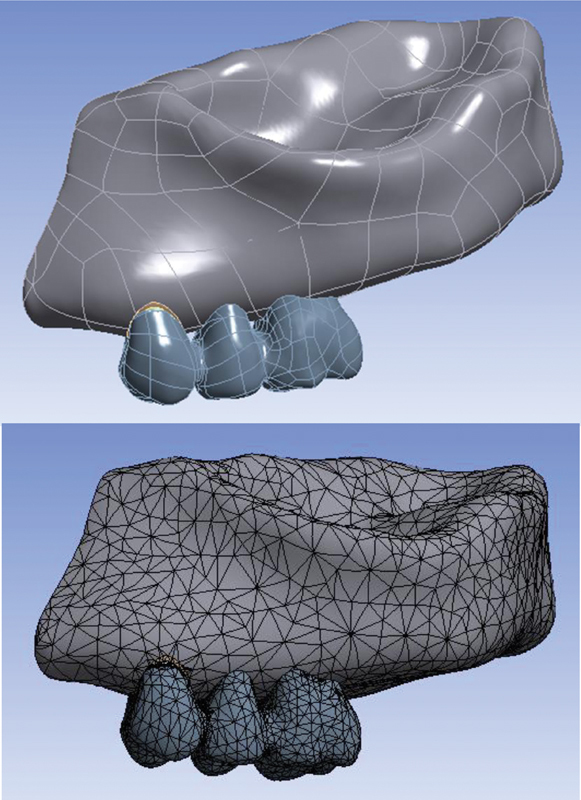
Analysis in ANSYS software. The total number of elements in the model was equal to 93,991 tetrahedral elements and the number of nodes was equal to 172,064.

**Appendix H:**
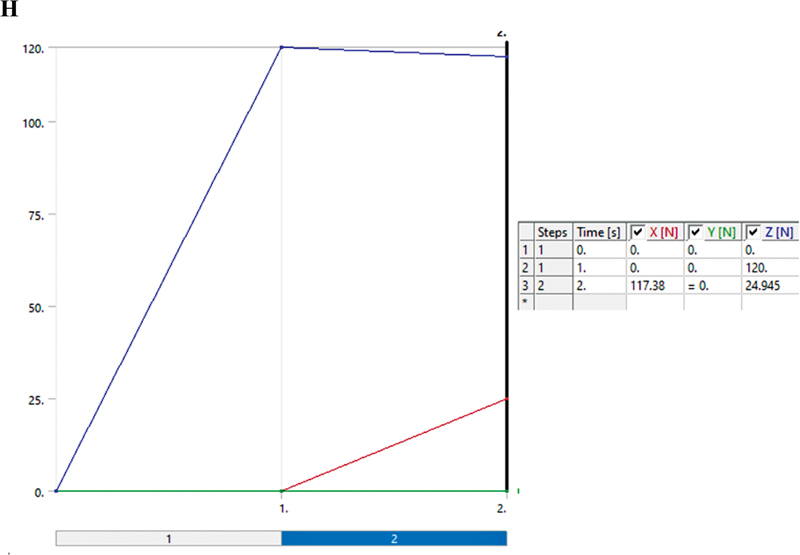
Boundary conditions for group function occlusal scheme.

**Appendix I:**
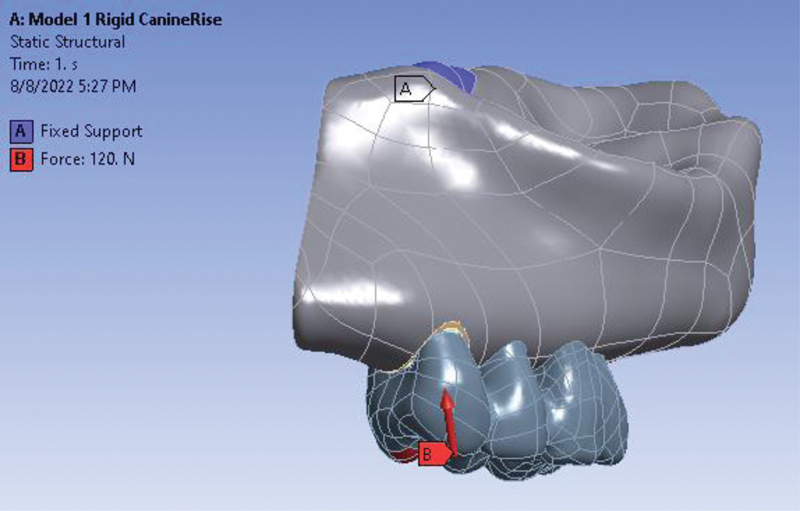
Boundary conditions for group function occlusal scheme and loading force detail.

### Analysis of ANSYS Software:2

After converting all geometries into .stp format, these geometries were entered into FEA (ANSYS software, Ansys 19.1; Ansys Inc; Canonsburg, Pennsylvania, United States). The three models were then meshed in the software and the mechanical properties of the materials were also uploaded for stress analysis. Finally, each designed model was analyzed with occlusal schemes, named group function ( Appendix G ).

#### Boundary Conditions for Group Function Occlusal Scheme

Loads equal to 120 N, 120 N, and 180 N were applied to the first premolar, second premolar, first molar, and second teeth, respectively, to simulate the group function occlusal scheme. The loads were first applied vertically, and then with 12-degree angulation. The upper surface of the maxilla was fixed ( Appendixes H and I ).

There were 93,991 tetrahedral elements and 172,064 nodes in the models. The elasticity modulus and Poisson's ratio of the pieces were defined according to the literature ( Table 1 ). The components were considered to be homogenous, isotropic, and linear.

**Table 1:** Materials' elasticity modulus ( *E* ) and Poisson's ratio ( *v* )

Material properties	Elasticity modulus ( *E* ) (GPa)	Poisson proportion ( *v* )
Dentin	18.6	0.31
Implant	110	0.33
Cortical bone	15	0.30
Ni–Cr alloy	218	0.33
Enamel	84	0.33
Periodontal membrane	2	0.45
Porcelain	69	0.28
Spongiose bone	1.5	0.30
Nonrigid attachment	110	0.33
Zinc phosphate cement	22.4	0.35

The models were analyzed by Ansys 2020 software, and the applied stress to the components and the total deformation (vertical displacement) of the natural teeth were measured and converted into color graphics.

The constructed numerical models of the study are verified by the previous study. 27

## Results

**Fig. 1:**
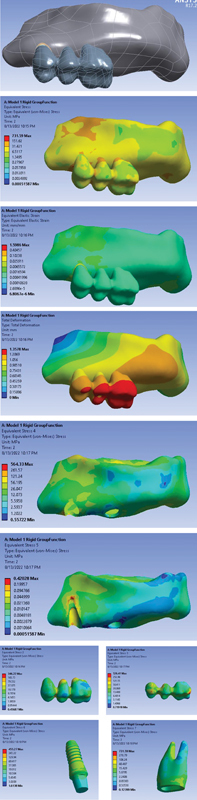
FPD with RC and group function occlusal occlusion ( **A** ) FPD model; ( **B** ) von Mises stress distribution in the entire model; ( **C** ) strain distribution in the entire model; ( **D** ) total deformation in the entire model; ( **E** ) von Mises stress distribution in the cortical bone; ( **F** ) von Mises stress distribution in the sponge bone; ( **G** ) von Mises stress distribution in FPD crown; ( **H** ) von Mises stress distribution in metal frame work; ( **I** ) von Mises stress distribution in implant; ( **J** ) von Mises stress distribution in dentin. FPD, fixed partial denture.

**Fig. 2:**
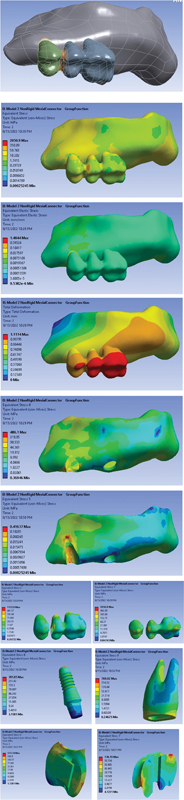
FPD with NRC in mesial and group function occlusion ( **A** ) FPD model; ( **B** ) von Mises stress distribution in the entire model; ( **C** ) strain distribution in the entire model; ( **D** ) total deformation in the entire model; ( **E** ) von Mises stress distribution in the cortical bone; ( **F** ) von Mises stress distribution in the sponge bone; ( **G** ) von Mises stress distribution in FPD crown; ( **H** ) von Mises stress distribution in metal frame work; ( **I** ) von Mises stress distribution in implant; ( **J** ) von Mises stress distribution in dentin; ( **K, L** ) von Mises stress distribution in connector. FPD, fixed partial denture; NRC, nonrigid connector.

**Fig. 3:**
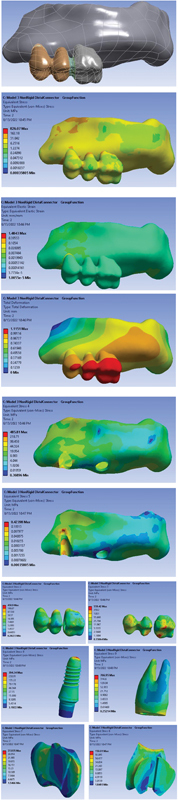
FPD with NRC in distal and group function occlusion ( **A** ) FPD model; ( **B** ) von Mises stress distribution in the entire model; ( **C** ) strain distribution in the entire model; ( **D** ) total deformation in the entire model; ( **E** ) von Mises stress distribution in the cortical bone; ( **F** ) von Mises stress distribution in the sponge bone; ( **G** ) von Mises stress distribution in FPD crown; ( **H** ) von Mises stress distribution in metal frame work; ( **I** ) von Mises stress distribution in implant; ( **J** ) von Mises stress distribution in dentin; ( **K, L** ) von Mises stress distribution in connector. FPD, fixed partial denture; NRC, nonrigid connector.

Three models of different connectors (rigid, nonrigid mesial connector, and rigid distal connector) were stimulated and are described as:

Model 1: A three-unit PFM bridge in which an RC is located on the implant side. The applied stress to the different parts of the model under vertical and oblique loading is depicted in Fig. 1 .
Model 2: A three-unit PFM bridge in which an NRC is located on the mesial side (tooth side). The applied stress to the different parts of the model under vertical and oblique loading is displayed in Fig. 2 .
Model 3: A three-unit PFM bridge in which an NRC is located on the distal side (implant side). The applied stress to the different parts of the model under vertical and oblique loading is displayed in Fig. 3 .

von Mises stress and total deformation distribution of the models under vertical and oblique loading are shown in Figs. 1 2 to 3 subsequently. As shown in the figures, the stress is highest in NRCs (shown with red color). The stress in dentin is 731.59 in the rigid connector, while it is 769.02 in nonrigid mesial connector and 766.95 for the distal connector. The average stress is significantly minimized by rigid connectors. Only the average stress for implants is higher in comparison with NRCs ( Table 2 ).

**Table 2:** von Mises stress

Stress, MPa		Part
346.22	Rigid	Crown
1172.9	Nonrigid mesial	
450.9	Nonrigid distal	
526.41	Rigid	Metal frame
2050.9	Nonrigid mesial	
559.42	Nonrigid distal	
564.33	Rigid	Cortical
486.1	Nonrigid mesial	
485.81	Nonrigid distal	
0.42	Rigid	Spongy
0.416	Nonrigid mesial	
0.423	Nonrigid distal	
455.27	Rigid	Implant
395.85	Nonrigid mesial	
394.34	Nonrigid distal	
731.59	Rigid	Dentin
769.02	Nonrigid mesial	
766.95	Nonrigid distal	
–	Rigid	Connector
354.76	Nonrigid mesial	
88.92	Nonrigid distal	

## Discussion

The present biomechanical study set out with the aim of assessing the importance of using NRCs and their location in the success of the partial TIFP. Previous studies used vital teeth instead of endodontically treated teeth and have shown controversial results. This article investigates the stress in the endodontically treated tooth in the maxillary posterior region.

Based on our findings, there is less stress in the crown, metal frame, and dentin when using rigid connectors in comparison to NRCs. Furthermore, the average stress in the crown, metal frame, and dentin is less in distal NRCs in comparison to mesial NRCs. Using an NRC led to less stress in the implant and cortical bone. The average stress in the implant is highest when using rigid connectors and lowest in mesial connectors. The applied stress was approximately equal in spongy bone.

Implant and natural tooth react differently toward stress. Natural tooth will intrude due to the presence of periodontal ligament, whereas implant is ankylosed to surrounding bone. 4 This difference may cause bending stress between the implants and the prosthesis. Several studies suggested the use of NRC to reduce the stress on implant, teeth, and surrounding structures. 22 28 29 Mosharraf et al supported the idea that NRC can act as a stress-absorbing structure and reduce the stress in implant and prosthesis. 30 This idea was also supported by several studies. 22 28 29 Rangert et al stated that the load was properly distributed between the implant and the tooth through the inherent bending flexibility of the implant screw joint. 29 Bechelli and Huang et al supported the role of NRC as stress absorbers. 22 28 Mosharraf et al reported that using NRC significantly decreases the applied stress to the prosthesis, implant, natural tooth, and bone in the anterior region of the maxilla. However, this opposed our finding, this conflict may be due to the difference in loading and anatomical structure between the anterior and posterior regions of the maxilla and the difference between natural and endodontically treated teeth. Natural teeth, gutta-percha, and post materials have different physical characteristics, and it has been established that resistance of a tooth to fracture is strongly corelated with remaining structure of the tooth and the elastic modulus of the post. 26 Furthermore, they have reported that NRC on the tooth side is more effective than using NRC on the implant side which is also in contrast to our findings. Lin et al suggested that the role of NRC as stress breakers is beneficial when the tooth is vital. But for the endodontically treated tooth, a rigid connector is more beneficial, this confirms our finding in using a rigid connector for endodontically treated tooth. 31 It was also suggested that the NRC minimizes the stress under the axial force. Therefore, NRCs are useful in the anterior region where the force distribution is axial. 32 Hamed and Mously reported that maximum stress was more in NRC, and the length and diameter of the implant are the main factor in reducing stress application. 33 This idea is supported by other studies. 34 35 It was reported in previous studies that the stress applied to natural tooth is always less than the stress applied to implant 22 28 29 30 ; however, the result of the study does not support this idea. This is mainly because of the difference between natural tooth and tooth with root canal therapy and the difference in their characteristics. 26

Due to different structures of cortical and spongy bone, their difference in elastic modulus and rigidity of the cortical bone is more suspectable for stress accumulation. 20 Some studies reported that using NRC can reduce the stress in bone. 23 32 Our study confirms these findings. However, Melo et al did not observe any stress reduction in bone. 36

There is also controversy about the location of the NRC too. Our finding suggested that using NRC in distal (attached to implant) reduces stress. These findings have been confirmed by Ozçelik and Ersoy. 20 It was stated that having NRC on the implant side will reduce the torque effect of the implant and reduce the stress 20 ; however, they have stated that using an NRC will reduce the stress in comparison to the rigid connector which is in contrast to our findings. On the other hand, some studies suggested to use NRC attached to the tooth. Both of these studies investigated a vital tooth which has a higher fracture resistance and movement in comparison to endodontically treated tooth. 4 27 Also, Koosha and Mirhashemi suggested that there is no difference in stress in supporting structures between the mesial and distal NRC, because the rotational center of an implant is higher than tooth, there is greater implant displacement when the NRC is attached to implant. 23

For effective calculation, the material properties of this study were assumed to be homogeneous, isotropic, and linear elastic conditions, which did not completely conform to the real clinical conditions. Due to these limitations, the results obtained in this study may not be exactly the same as the actual values, but it could reveal the difference in stress and displacement between groups to provide clinicians with judgments on the design of the prosthesis. The synergistic utilization of artificial intelligence and FEA studies holds the potential to unlock valuable insights into the practical applications of dental implants. 37 38 39 40 41 Further long-term clinical studies to examine the effectiveness of NRC are needed.

## Conclusion

Rigid connectors decreased the stress in comparison to nonrigid connectors. Furthermore, distal NRC was more efficient than mesial NRC. Using NRC will only reduce the stress applied to cortical bone. Therefore, it is recommended to use a rigid connector in the maxillary posterior region when the tooth is endodontically treated.
